# Impacts of crowding, trawl duration and air exposure on the physiology of stingarees (family: Urolophidae)

**DOI:** 10.1093/conphys/cou040

**Published:** 2014-10-03

**Authors:** Matthew Heard, Jason A. Van Rijn, Richard D. Reina, Charlie Huveneers

**Affiliations:** 1 School of Biological Sciences, Flinders University, Sturt Road, Adelaide, South Australia 5042, Australia; 2 School of Biological Sciences, Monash University, Wellington Road, Clayton, Victoria 3800, Australia

**Keywords:** Air exposure, blood chemistry, delayed mortality, granulocyte-to-lymphocyte ratio, post-release, trawl capture

## Abstract

Research on physiological stress and post-capture mortality of threatened species caught as bycatch is critical for the management of fisheries. The present study used laboratory simulations to examine the physiological stress response of sparsely spotted stingarees (*Urolophus paucimaculatus*) subjected to one of four different trawl treatments, including two different trawl durations as well as ancillary stressors of either air exposure or crowding. Physiological indicators (plasma lactate, urea, potassium and glucose) and changes in white blood cell counts were measured from blood samples taken throughout a 48 h recovery period. Mortality was low throughout this study (15% overall) and occurred only after >48 h following air exposure, crowding and 3 h trawl simulations. Plasma lactate, glucose and urea concentrations were identified as potential indicators of physiological stress, while plasma potassium and white blood cell counts were too variable to identify changes that would be expected to have biological consequences for stingarees. The characterization of the temporal profiles of physiological indicators facilitates a more accurate assessment of secondary stressors by identifying the best timing to sample stingaree blood when investigating post-capture stress physiology. High levels of lactate, increasing glucose and depressed urea were all recorded in response to air exposure following trawling, indicating that this is the primary source of stress in stingarees caught in trawling operations. These findings highlight the importance of improving bycatch sorting procedures to reduce the time out of the water for trawl-caught stingarees.

## Abstract (precis)

Stingarees were trawled under controlled laboratory conditions to investigate levels of mortality from trawling, the individual stressors involved and the physiological indicators that may predict stress. Low levels of mortality were recorded with lactate and urea promising indicators of stress. And air exposure inducing the greatest stress response in stingarees.

## Introduction

With bycatch estimated to be approximately a quarter of the world fisheries catch, mortality of non-targeted species has become a critical problem for fisheries management worldwide (Davis, 2002). Towed fishing gears (e.g. Danish seines, otter and beam trawls and dredges) comprise more than half the total global catch but are highly non-selective fishing methods that result in rates of bycatch up to three times the target catch (Broadhurst *et al.*, 2006; Gillet, 2008). Although numerous studies report levels of mortality at the time of capture and the amounts of live fish being discarded (see Broadhurst *et al.*, 2006), a comprehensive understanding of the effects of post-capture stress on the survival rates and physiological state of bycatch is still lacking (Davis, 2002; Cooke and Schramm, 2007; Braccini *et al.*, 2012).

The impact of a capture event on an individual animal is influenced by a range of biotic and abiotic variables that can be specific to the individual (e.g. size, age, maturity and degree of physical damage) or to the type of capture event (e.g. gear type, capture duration, rapid changes in temperature and pressure and handling procedures; Davis, 2002; Skomal, 2007; Frick *et al.*, 2010a, b; Braccini *et al.*, 2012; Skomal and Mandelman, 2012; Wilson *et al.*, 2014). Air exposure has been identified as a significant contributor to capture stress in teleosts (Davis, 2002) and more recently in elasmobranchs (Frick *et al.*, 2010b; Cicia *et al.*, 2012), inferred from the accumulation of metabolites in the blood, the induction of intracellular acidosis, and osmotic and ionic imbalances (Cicia *et al.*, 2012).

Physiological research can provide a basis for quantifying stress and predicting an individual's probability of survival following capture in fishing gear, handling and discard by fishers (Barton, 2002; Walker, 2007; Skomal and Mandelman, 2012). Acute stress in elasmobranchs, such as that due to fisheries capture, often results in changes in blood chemistry as energy stores (e.g. glucose) are mobilized, ion balances are disrupted and metabolites (e.g. lactate and urea) move from the muscle cells into the bloodstream (Bonga, 1997; Skomal and Mandelman, 2012). Consequently, physiological indicators of stress are commonly measured through the analysis of blood, with samples taken immediately after capture representing the initial capture response, and subsequent readings providing a profile to assess the individual's ability to recover from stress over time (Marcalo *et al.*, 2006; Skomal and Benrnal, 2010; Frick *et al.*, 2012).

Differential leucocyte counts have been used to provide insight into the response of an elasmobranch immune system to defend against infection commonly associated with stress of capture (Davis *et al.*, 2008; Van Rijn and Reina, 2010). Lymphocytes are responsible for the production of antibodies and cell-mediated immunity, while granulocytes (heterophils and neutrophils) are the most active phagocytic and pinocytic cells, which generally increase in response to infection, disease and stress (Semeniuk *et al.*, 2009). An increase in the granulocyte-to-lymphocyte (G/L) ratio can occur within 24 h of stress (Van Rijn and Reina, 2010) and reflects a change in immune strategy, which is indicative of longer-lasting impacts of stress resulting from a capture event (Davis *et al.*, 2008).

Elasmobranchs, particularly benthic species such as stingarees (family Urolophidae), generally have a low metabolic rate (in comparison to teleost fishes) and have previously been shown to exhibit a slow metabolic response to stress (Davis, 2002; Frick *et al.*, 2009, 2012). In these species, physiological indicators of stress may not peak until hours after a stressful event, making elasmobranchs more likely to succumb to post-capture mortality caused by the accumulation of harmful metabolic byproducts at a later stage than teleost species (Frick *et al.*, 2009). Therefore, it is important that studies measure the physiological performance of elasmobranchs for hours or days following a capture event in order to assess the response of an elasmobranch to a stressor accurately (Frick *et al.*, 2009). The majority of field studies measure physiological parameters only at the time of capture and may not be able to characterize the stress response of some elasmobranchs completely. Controlled experiments conducted in the laboratory environment increase our understanding of the physiological response to fishing capture in elasmobranchs and further elucidate the relationship between elasmobranch stress physiology and post-capture mortality (Wood, 1991; Frick *et al.*, 2010b; Brooks *et al.*, 2012).

Demersal trawling has been recognized as one of the major threats to the survival of many demersal red-listed elasmobranch species (Last and Stevens, 2009). This is likely to be related to the life-history characteristics displayed by many elasmobranch species (e.g. slow growth, late maturity, low fecundity), which make them particularly susceptible to overfishing (Hoenig and Gruber, 1990; Musick *et al.*, 2000). The family Urolophidae presently contains 28 species, of which five are listed as Threatened by the IUCN, three as Vulnerable (*Urolophus viridis*, *Urolophus sufflavus* and *Urolophus bucculentus*), one as Endangered (*Urolophus orarius*) and one as Critically Endangered (*Urolophus javanicus*; see www.iucnredlist.org). Urolophids are a major component of the elasmobranch bycatch caught by the demersal trawl fisheries that operate in Australian waters and are discarded due to their low commercial value, but very little is currently known of their post-capture survival (Last and Stevens, 2009; Trinnie *et al.*, 2009). Studies on the composition of bycatch in the Southern and Eastern Scalefish and Shark Fishery and the Spencer Gulf Prawn Fishery have indicated that the level of bycatch may not be sustainable for some stingarees (Thomas and Chick, 2007; Walker and Gason, 2007; Trinnie *et al.*, 2009). In addition to the uncertainty about the bycatch mortality of stingarees, this family is highly susceptible to capture-induced abortion, which further compounds the impacts of a capture event (White and Potter, 2005; Trinnie *et al.*, 2009).

The aim of this study was to examine, in controlled laboratory conditions, the effects of different stressors involved in trawl capture (trawl duration, air exposure and crowding) on the physiological response of a benthic elasmobranch species. We aimed to replicate the conditions experienced by stingarees during standard commercial trawls and used trawl and air-exposure times that reflected trawl and handling times in the South Australian commercial prawn fisheries in which *Urolophus* spp. are regular bycatch (Thomas and Chick, 2007). Physiological response was measured over a 2 day sampling and monitoring period through the analysis of blood metabolites, ions and leucocyte counts, because these have previously been identified as suitable capture stress indicators (Skomal and Mandelman, 2012), with G/L ratio being suggested as a potential indicator of the tertiary stress response in elasmobranchs (Davis *et al.*, 2008; Van Rijn and Reina, 2010). Specifically, we aimed to determine: (i) the mortality rate of stingarees in an experimental trawl; (ii) the physiological responses most likely to indicate stress in stingarees; and (iii) the individual stressors involved in a capture event likely to cause physiological stress and mortality of stingarees.

## Materials and methods

### Ethics statement

This research was conducted under the Flinders University Animal Welfare ethics permit E288. Animal collection and release was authorized by the Victorian Department of Primary Industries under the general research permit RP 983 and Victorian ministerial approval AW/00273.

### Animal collection and husbandry

**Figure 1:**
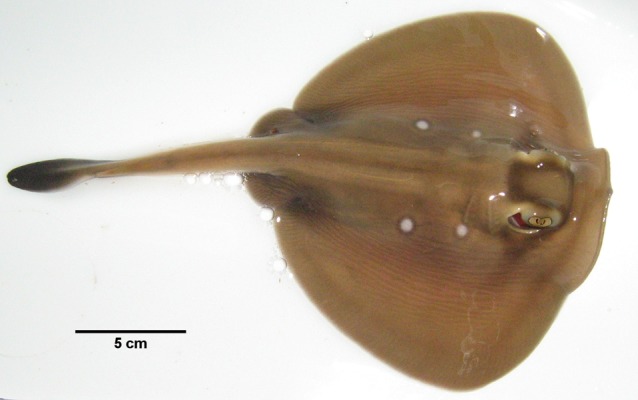
Study species. Sparsely spotted stingaree (*Urolophus paucimaculatus*).

Stingarees (*Urolophus paucimaculatus*; Fig. 1) were caught by a commercial fisher in Port Philip Bay (Victoria, Australia) using beach seine nets in October 2009. Stingarees were transported to the Marine and Freshwater Fisheries Research Institute in Queenscliff (Victoria, Australia) in a 1000 l sealed tank, filled with ambient seawater and mounted on a car trailer. The transport time between collection and housing did not exceed 2 h. Prior to transferring stingarees into the housing tanks, total length (TL) was measured to the nearest 0.5 cm.

Animals were housed at a density >750 l per individual in large circular tanks connected to a flowthrough ambient seawater system and separate air lines for a period of at least 10 days prior to experimentation. They were fed a diet of chopped prawns to satiation three times a week but remained unfed for 4 days prior to experimentation to prevent food metabolism from affecting physiological indicators. Water quality was monitored on a daily basis for temperature and oxygen concentration, with the ambient water temperature ranging from 13.7 to 17.6°C and the oxygen concentration ranging from 93 to 103% saturation over the 2 month duration of the study.

### Control

Control animals (*n* = 8 animals; mean ± SD TL, 33.62 ± 3.66 cm) were used to determine the effects of repeated handling and blood sampling and were not put through any trawling treatments, but were subjected to identical handling procedures to the treatment animals. An identical repeated blood-sampling procedure was followed for both control and experimentally trawled animals, with the initial blood samples from the control group providing baseline blood samples.

### Trawling experiments

For trawling experiments, each animal was transferred by dip net from its holding tank to a trawl cod-end (monofilament mesh, 10.2 cm diameter, 110 cm depth), with its ventral side on the mesh. The trawl cod-end was then placed in a 19 000 l experimental tank previously described by Frick *et al.* (2010b). A large (100 cm high × 200 cm wide) paddle in the centre of this tank, driven by a 240 V three-phase motor, circulated the water to create a water current of ∼0.6 m/s in front of the cod-end. Stingarees exposed to the trawling treatment were assigned to one of four different treatment groups designed to compare the impacts of different stressors of trawl capture.

The four treatments were as follows.

- A trawling time of 1 h (*n* = 8 animals; mean ± SD TL, 34.25 ± 2.49 cm) was selected as the standard trawling time because it represents the common trawling time used in the South Australian Prawn Trawl Fisheries (Dixon *et al.*, 2013).
- A trawling time of 3 h (*n* = 8 animals; mean ± SD TL, 33.38 ± 1.92 cm) was selected to assess the impacts of longer trawl durations occasionally used in the South Australian Prawn Trawl Fisheries.
- The impact of air exposure during the time taken to discard the catch (*n* = 8 animals; mean ± SD TL, 34.87 ± 2.85 cm) was tested using a 1 h trawl followed by 10 min of air exposure in a fish crate.
- The impact of crowding (*n* = 10 animals; mean ± SD TL, 33.25 ± 2.71 cm) was tested by placing five stingarees into the cod-end at the same time for 1 h.

Stingarees were put into the cod-end individually for all treatments except the crowded trawl treatment. Each animal was used for only a single experiment and was inspected by a veterinarian before being returned into Port Phillip Bay. Stingarees that died following treatments are referred to as ‘moribunds’ and stingarees that survived as ‘survivors’.

### Blood sampling

For each blood sample, stingarees were placed in a small tub, partly filled with water to ensure that the gills remained submerged, with their ventral side up and restrained using a gloved hand. Blood samples of ∼0.5 ml were taken using the caudal venipuncture technique with a 23 gauge hyperdermic needle attached to a 1 ml sterile syringe, which had been pre-treated with sodium heparin. Blood samples were taken immediately after treatment (time 0) and 2, 6, 24 and 48 h post-treatment. The time taken to obtain each blood sample (*n* = 200; mean ± SD 91.4 ± 38.3 s) did not exceed 3 min for any sample. Samples were centrifuged at 12 879 *g* for 8 min prior to removing the plasma from the primary sample, and plasma was stored at −20°C for later analysis. Blood plasma concentrations of lactate, glucose, urea and potassium were analysed at the Institute for Medical and Veterinary Science at Flinders Medical Centre (Adelaide, Australia).

### Leucocyte profiles

Prior to centrifuging the blood samples, two blood smears were made using whole blood to determine changes in leucocyte populations. Blood smears were allowed to air dry in sealed containers for between 12 and 24 h before fixing in methanol for 10 min. Fixed slides were stained with May-Grünwald (solution diluted 1:1 with water; Australian Biostain, Traralgon, Victoria, Australia) and Giemsa (solution diluted 1:9 with water; Australian Biostain) for 15 min each before rinsing three times in water and standing in distilled water for 5 min. Slides were examined using compound microscopes at ×400 magnification in areas of even cell spread and consistent cell integrity. Leucocyte counts were made using the method described by Van Rijn and Reina (2010). A minimum of 200 granulocytes (neutrophils, heterophils and eosinophils) and lymphocytes were identified per slide, and counting ceased only when all cells in the final field of view were identified. Two hundred cells were counted to increase the accuracy of the count and to decrease the variance caused by heterogeneous cell distribution on the slide (Van Rijn and Reina, 2010).

**Figure 2:**
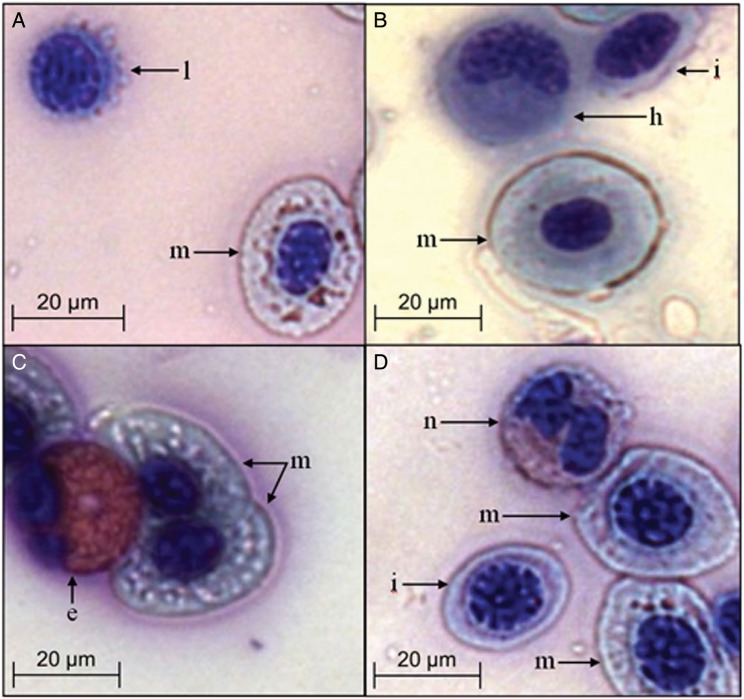
Blood cell morphology in sparsely spotted stingarees (*U. paucimaculatus*). (**A**) Lymphocyte (l) and mature erythrocyte (m). (**B**) Immature erythrocyte (i), heterophil (h) and mature erythrocyte (m). (**C**) Mature erythrocytes (m) and eosinophil (e). (**D**) Neutrophil (n), mature erythrocytes (m) and immature erythrocyte (i).

All erythrocytes (red blood cells) and leucocytes were classified using the criteria of Clauss *et al.* (2008). Mature erythrocytes were oval to elliptical in shape, with a conspicuous centric nucleus and a low nucleus-to-cytoplasm ratio, while immature erythrocytes were smaller, more rounded and with a higher nucleus-to-cytoplasm ratio (Fig. 2). Lymphocytes were identified as cells with densely basophilic (blue) nuclei, high nucleus-to-cytoplasm ratio and often blebs on the outer membrane (Fig. 2A). Heterophils had heavily granulated basophilic cytoplasm, with an eccentric rounded nucleus (Fig. 2B). Eosinophils were identified through distinct bright pink eosinophilic granulated cytoplasm and eccentric nuclei (Fig. 2C). Neutrophils had highly granulated, slightly eosinophilic (light pink) cytoplasm and distinct eccentric nuclei that were commonly lobed or segmented (Fig. 2D).

Leucocyte changes occur slowly in response to stress in ectotherms (Davis *et al.*, 2008). To determine the speed of these changes, we initially counted the 0, 24 and 48 h samples for the air-exposure group. As the change from 0 and 48 h samples was much greater than the change from 0 to 24 h samples, we subsequently counted the blood smears for the 0 and the 48 h samples for the control and the 1 and 3 h trawl treatments.

The accuracy of the leucocyte profiles was tested for each count by directly calculating the percentage difference between the total number of granulocytes and lymphocytes in the first 100 cells compared with the second 100 cells. Two investigators conducted blood smear counts, with variance between investigators tested by comparing the total number of granulocytes and lymphocytes counted by each investigator. Variance between investigator counts was found to be <10% through a comparative test on 36% of blood smear slides. For all analyses, the number of granulocytes was calculated by summing the number of neutrophils and heterophils counted. All leucocyte profiles are presented as G/L ratios and percentage change to remain consistent with previous literature (see Davis *et al.*, 2008).

### Statistical analysis

The Statview program (SAS Institute Inc.) was used to compare survival in all treatment groups using a logrank Mantel–Cox analysis of the χ^2^ survival statistic. All other statistical analysis was conducted using PASW Statistics 18 (IBM SPSS Inc.). Differences in the length of stingarees between treatment groups and between males and females were analysed using one-way analysis of variance (ANOVA). Pearson's χ^2^ test was used to compare the ratio of males and females in the treatment groups. The effect of the time taken to obtain the blood sample on the lactate concentration was tested using Pearson's correlation. We used lactate for this correlation because it has previously been shown to have a faster response time to stress in elasmobranchs than other blood variables (Frick *et al.*, 2010a).

Changes in blood variable concentrations and G/L ratios over time and between treatments were analysed using linear mixed-effects models, with repeated-measures covariance hypothesized to be autoregressive heterogeneous and restricted maximum likelihood estimation used, which allowed us to incorporate measurements from animals with some missing samples (Wang and Goonewardene, 2004). *Post hoc* pairwise comparisons with Bonferroni correction were used to investigate differences between treatments for blood variable concentrations when there was no significant interaction between treatment and time. At each sampling time, differences in blood variable concentrations and G/L ratios between treatments were assessed by presenting mean concentrations with 95% confidence intervals (CIs) as a way of reporting analyses with significant interactions (Cumming *et al.*, 2007; Beninger *et al.*, 2012). This approach was chosen because corrections for testing multiple comparisons were considered too conservative to assess the large number of comparisons required for this data set (Perneger, 1998; Garcia, 2004). Significant differences were determined at *P* < 0.05. All results are presented as a mean ± standard error unless otherwise stated.

## Results

### Observations and mortality

Forty stingarees (33.87 ± 0.42 cm TL) were collected and held within the seawater facilities at MAFFRI for 27 days. Females (*n* = 22; 34.54 ± 0.66 cm TL) were slightly more numerous and larger than males (*n* = 18; 33.05 ± 0.44 cm TL), but were not significantly different in size compared with males (ANOVA, *F*_1,38_ = 0.07, *P* > 0.05). There was no significant difference in the TL (ANOVA, *F*_4,35_ = 0.48, *P* > 0.05) or sex (Pearson's χ^2^,χ_4_^2^=3.64, *P* > 0.05) of stingarees between treatment groups.

During trawling simulations, water flow in the tank prevented stingarees from swimming freely inside the cod-end, and >90% of the stingarees remained with their ventral side into the net for the duration of the trawl. When removed from the cod-end at the end of the trawl, stingarees generally struggled for about 5–10 s before a blood sample could be taken. There was negligible correlation between blood-sampling time (*n* = 200, 91.4 ± 2.7 s) and blood lactate levels (Pearson's correlation: *n* = 191, *r* = 0.018, *P* > 0.05). During the air-exposure treatment, stingarees generally remained motionless throughout the 10 min of air exposure, but short bouts of struggling were occasionally observed.

The number of granulocytes (Student's paired *t*-test: *t*_12_ = 1.45, *P* > 0.05) or lymphocytes (Student's paired *t*-test: *t*_12_ = 0.15, *P* > 0.05) counted by each investigator was not different on slides that were recounted to measure consistency between investigators. We observed a difference of 20.38 ± 1.74% in the G/L ratio between the first and second 100 cells counted on each slide, but this is not considered to be clinically (Gross and Siegel, 1983) or statistically significant (Student's paired *t*-test: *t*_65_ = 0.24, *P* > 0.05).

Mortality did not occur in the control group or while any of the trawling treatments were taking place. Instead, all mortality occurred between 48 and 96 h post-capture, with three deaths occurring between 48 and 72 h and three deaths between 72 and 96 h. The overall mortality rate was 15%, with three deaths occurring in the 3 h trawl group, two deaths in the crowded treatment group, and one death in the air-exposure group. There was no significant difference in survival when comparing all groups (χ_4_^2^=2.3, *P* = 0.68) or only the control and 3 h trawl groups (χ_1_^2^=1.42, *P* = 0.23).

### Physiological indicators of capture stress

#### Control

**Figure 3:**
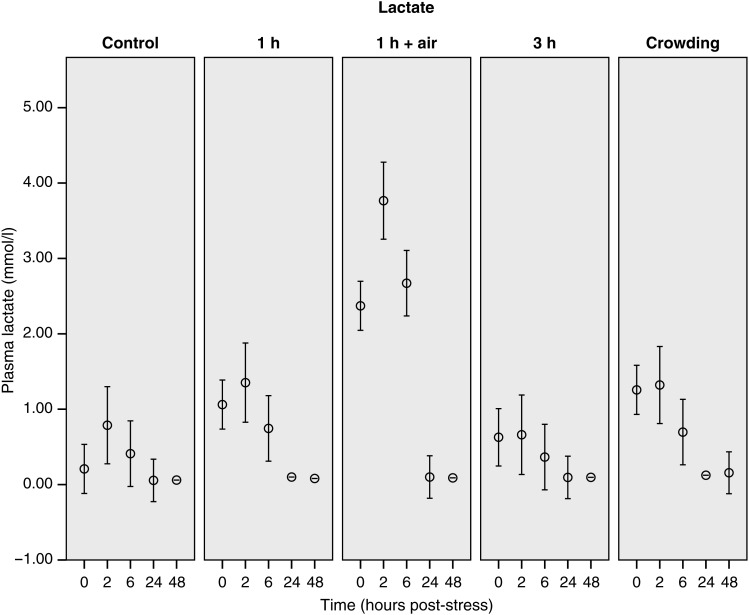
Changes of lactate concentrations of stingarees (*U. paucimaculatus*) post-capture. Mean [±95% confidence interval (CI)] plasma lactate concentration of stingarees over a 48 h recovery period following varying trawl exposures in captivity.

**Figure 4:**
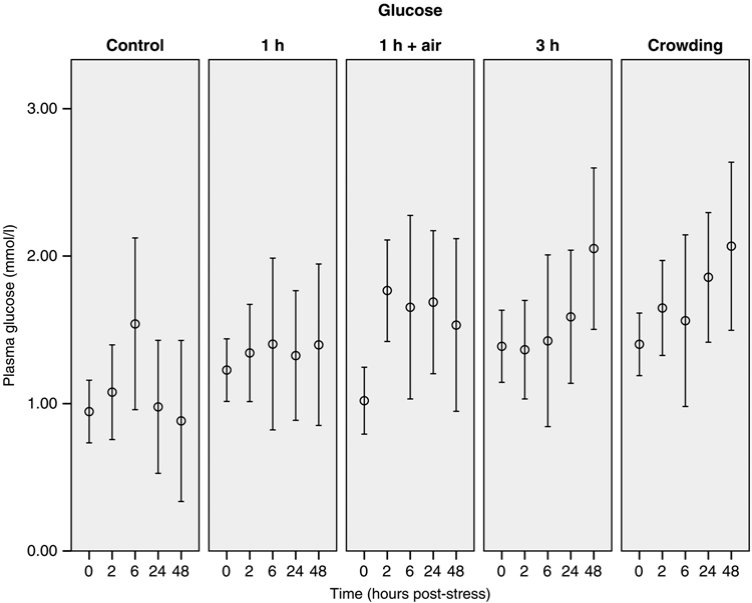
Changes of glucose concentrations of stingarees (*U. paucimaculatus*) post-capture. Mean (±95% CI) plasma glucose concentration of stingarees over a 48 h recovery period following varying trawl exposures in captivity.

**Figure 5:**
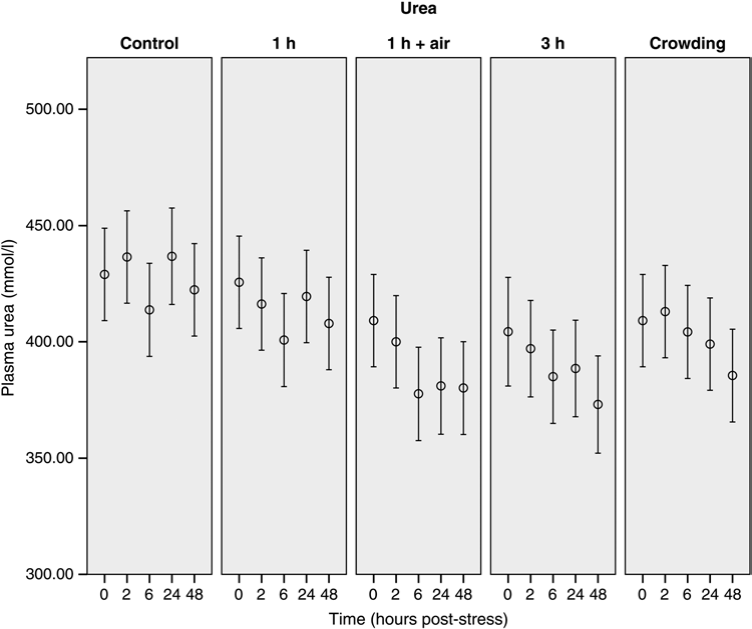
Changes of urea concentrations of stingarees (*U. paucimaculatus*) post-capture. Mean (±95% CI) plasma urea concentration of stingarees over a 48 h recovery period following varying trawl exposures in captivity.

**Figure 6:**
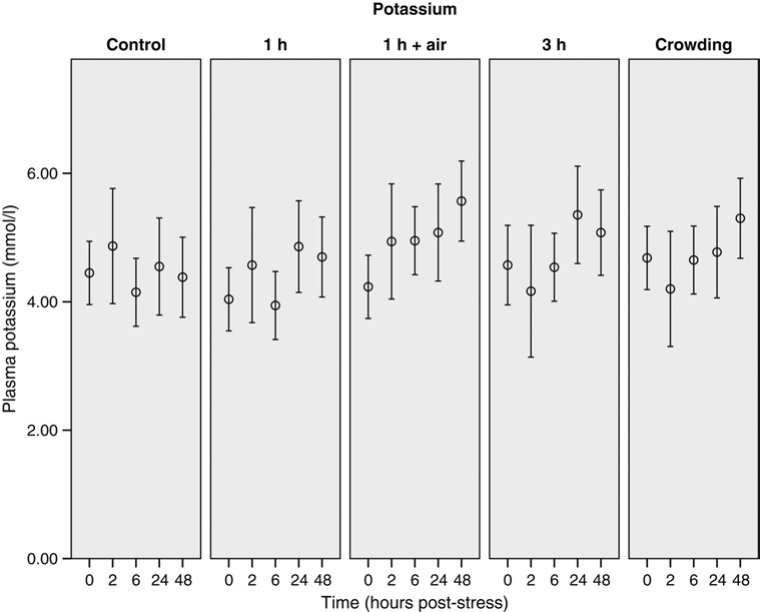
Changes of potassium concentrations of stingarees (*U. paucimaculatus*) post-capture. Mean (±95% CI) plasma potassium concentration of stingarees over a 48 h recovery period following varying trawl exposures in captivity.

**Figure 7:**
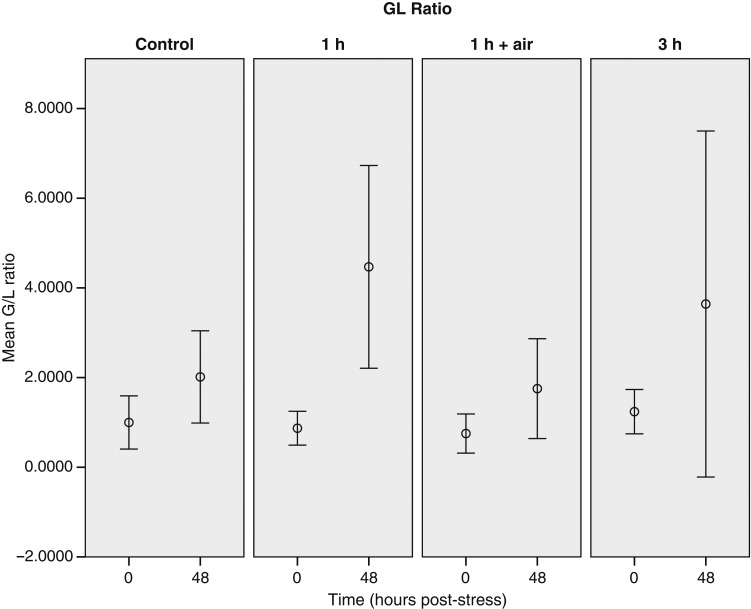
Changes of granulocyte-to-lymphocyte (G/L) ratio of stingarees (*U. paucimaculatus*) post-capture. Mean (±95% CI) G/L ratio of stingarees over a 48 h recovery period following varying trawl exposures in captivity.

The 0 h sample of the control group is considered to represent baseline (i.e. unstressed) values for this species (Table 1). Only minor changes in the measured plasma concentrations (lactate, urea, potassium and glucose) were observed over the blood-sampling period in control animals (Figs 3–6). Additionally, there was no major change in G/L ratio from the 0 h sample (0.99 ± 0.23) to the end of the 48 h sampling period (2.02 ± 0.42; Fig. 7). We are confident that blood sampling and handling did not have an influence on the physiological indicators or G/L ratios recorded in this study.

**Table 1:** Mean (±SEM) plasma constituent concentrations and granulocyte-to-lymphocyte (G/L) ratios for treatment groups at initial sampling time (0 h) and maximal recorded lactate and G/L ratios 48 h post-capture, with percentage change over the 48 hour recovery period (mean ± SEM)

		Lactate (mmol/l)						G/L ratio		
Treatment	*n*	0 h	Maximum	Glucose (mmol/l) 0 h	Urea (mmol/l) 0 h	Potassium (mmol/l) 0 h	*n*	0 h	48 h	Percentage change
Control	8	0.21 ± 0.07	0.79 ± 0.35	0.95 ± 0.13	429.00 ± 9.99	4.45 ± 0.28	6	0.99 ± 0.23	2.02 ± 0.42	197.19 ± 33.88
1 h	8	1.06 ± 0.25	1.39 ± 0.41	1.23 ± 0.10	425.63 ± 5.62	4.04 ± 0.20	7	0.87 ± 0.16	4.47 ± 0.96	649.77 ± 102.38
3 h	8	0.05 ± 0.18	0.62 ± 0.32	1.33 ± 0.12	404.04 ± 13.19	4.57 ± 0.15	7	1.24 ± 0.21	3.64 ± 1.58	146.54 ± 35.17
1 h + air	8	2.37 ± 0.29	3.77 ± 0.59	1.02 ± 0.13	409.13 ± 7.48	4.23 ± 0.43	6	0.75 ± 0.18	1.75 ± 0.47	266.76 ± 30.18
Crowding	8	1.26 ± 0.24	1.32 ± 0.33	1.40 ± 0.13	409.13 ± 6.59	4.68 ± 0.17

#### Trawling treatments

Lactate concentration changed over the blood-sampling period (Table 2), peaking 2 h after the treatment before returning to baseline levels by 24 h post-treatment (Fig. 3). Plasma lactate was significantly different between treatments, and there was also an interaction between treatment and time (Table 2). Plasma lactate in air-exposed stingarees remained significantly elevated for the first 6 h of the sampling period compared with the control values and all other treatment groups based on non-overlapping CIs (Fig. 3). Elevated lactate levels were recorded immediately after trawl capture (0 h) in the air-exposure and crowded treatment groups when compared with the control values (Table 1).

**Table 2:** Linear mixed-model analysis of plasma constituent concentrations from experimentally trawled stingarees over a 48 h recovery period

	Lactate			Glucose			Urea			Potassium			G/L ratio		
Factors	d.f.	*F*	*P *value	d.f.	*F*	*P *value	d.f.	*F*	*P *value	d.f.	*F*	*P *value	d.f.	*F*	*P *value
Treatment	39.4	12.5	**0.00**	51.2	1.8	0.14	41.2	3.9	**0.01**	38.6	1.2	0.32	7.8	2.3	0.16
Time	44.7	34.3	**0.00**	37.4	5.9	**0.00**	64.6	6.0	**0.00**	62.7	3.8	**0.01**	17	19.8	**0.02**
Treatment × time	44.7	5.4	**0.00**	37.4	2.3	**0.02**	64.8	0.9	0.55	62.2	0.9	0.51	3.9	2.4	0.20

Bold values indicate a significant effect of treatment or time for the variable indicated, or a significant interaction between treatment and time.

Glucose concentrations changed over time (Table 2); however, initial glucose concentrations were not significantly different in the control group when compared with the trawl treatment groups (Table 1). Non-overlapping confidence intervals indicate that glucose concentrations were higher than the control group at 2 h post-capture in the air-exposure group and at 48 h post-capture in both the 3 h trawl and crowd treatments (Fig. 4).

Concentrations of urea changed over time and between treatments, but there was not a significant interaction between these two factors (Table 2). Pairwise comparisons showed significant differences in urea concentrations between the control and the air-exposure treatment (mean difference = 37.7 ± 6.9, d.f. = 136.4, *P* = < 0.001), the 3 h trawl (mean difference = 37.4 ± 7.1, d.f. = 139.5, *P* = < 0.001) and the crowd treatment (mean difference = 25.2 ± 6.8, d.f. = 135.9, *P* = 0.003). Plasma urea concentration decreased over the recovery period in the air-exposure and 3 h treatments and was significantly lower than in the control group at 24 and 48 h post-capture based on non-overlapping CIs (Fig. 5).

Potassium concentration exhibited changes over the blood-sampling period, but not between treatments (Table 2). Potassium concentrations increased through the sampling period in all treatment groups, with the greatest increase exhibited by the air-exposure treatment which was, at the end of the 48 h blood-sampling period, higher than that of the control stingarees (Fig. 6).

Like the potassium concentration, G/L ratios increased over the 48 h blood-sampling period but were not significantly different between treatments (Table 2). Some high values were recorded in the control group, but there was not a significant change from the 0 to the 48 h samples (Table 1). Overlap between the CIs of the G/L ratios recorded in the treatment groups and the control group at both the 0 and the 48 h sample indicate a lack of significant difference (Fig. 7); however, the high variability of G/L ratios between animals, particularly in the 48 h sample, might have prevented our ability to detect such changes. The greatest increase in G/L ratio was recorded in the 1 h trawl group, which increased from 0.87 ± 0.16 at the 0 h sample to 4.47 ± 0.96 at the 48 h sample, representing an increase of 649.77 ± 102.38% (Fig. 7).

## Discussion

The present study is the first investigation into the physiological stress response of stingarees in a controlled environment. Secondary stress indicators (lactate, glucose, potassium and urea) and tertiary indicators (G/L ratio) are the focus of this study due to the difficulties of measuring primary stress hormones in elasmobranchs raised by Pankhurst (2011). We acknowledge the difficulties of quantifying physiological changes using blood samples due to the constant exchange of metabolites and ions between blood, muscles and organs (Barton, 2002). Additionally, we recognize the limitations of secondary physiological indicators of stress, particularly plasma glucose, which has been shown to increase and decrease in response to stress in different fish species (Bonga, 1997). To overcome these limitations, multiple blood samples were collected and a suite of indicators has been used to investigate the impacts of stressors involved in capture.

Our results improve the understanding of the physiological changes of elasmobranchs recovering from stress. Through the collection of multiple blood samples following a capture event, this study provides further insight into specific metabolic processes induced by an acute stressor. Air exposure caused the greatest changes to plasma lactate concentrations over the first 6 h, but returned to baseline levels 24 h following treatment. Additionally, air exposure produced the greatest suite of changes across the parameters tested.

### Control

There were no significant changes in plasma concentrations (e.g. lactate, urea, potassium and glucose) of the control group over the blood-sampling period, suggesting that repeated blood sampling did not affect the blood parameters recorded and that the sampling method used was suitable. In addition, there was no correlation between the time taken to collect each blood sample and the plasma lactate concentration, which indicates that the time necessary to take the blood samples did not affect the stress levels of the stingarees.

Initial plasma lactate and glucose concentrations measured in the control group (0.21 ± 0.07 and 0.95 ± 0.13 mmol/l, respectively) are comparable to baseline levels reported in a previous study on physiological stress in sparsely spotted stingarees (0.41 ± 0.06 and 0.65 ± 0.05 mmol/l, respectively; Thomas and Chick, 2007). The resting plasma lactate, urea, potassium and glucose concentrations were also similar to the pre-stress values in Port Jackson sharks (*Heterodontus portusjacksoni*) and gummy sharks (*Mustelus antarcticus*; Frick *et al.*, 2010a).

### Mortality

Post-capture mortality (15% overall) occurred only after >48 h post-capture in the present study. This is much lower than the mortality rate of 94% for stingarees released into sea cages following commercial trawls as reported by Thomas and Chick (2007). The cumulative effects of the crowding, hypoxia and physical damage experienced by stingarees caught in that study may be more stressful than the experimental trawl conditions we were able to simulate. However, Thomas and Chick (2007) acknowledge that their results are inconclusive because the mortality in their study may have been caused by predation from sea lice.

When assessing post-capture mortality, laboratory simulations, such as those in this study, are not able to measure additional stressors following discard, such as predation or temperature changes (Davis, 2002). Post-capture mortality rates in commercial trawls may, therefore, be higher than those observed here.

### Physiological indicators of capture stress

#### Lactate

Plasma lactate concentration exhibited changes in response to crowding and air exposure, with maximal lactate concentrations recorded 2 h following trawl simulations and returning to baseline levels 24 h after stress. This is similar to patterns recorded in *Carcharhinus obscurus* (Cliff and Thurman, 1984), *H. portusjacksoni* (Frick *et al.*, 2010b) and *M. antarcticus* (Frick *et al.*, 2010a, 2012) and for *Chiloscyllium punctatum* and *Hemiscyllium ocellatum* (Chapman and Renshaw, 2009). The lactate peak at the 2 h sampling time rather than the initial (0 h) sample is an indication of the time required for lactate to move from the muscle cells into the bloodstream. This highlights the importance of characterizing the stress response through time, because peak lactate may not be evident directly after capture, potentially leading to an underestimation of stress and the physiological response.

In field studies of long-line-caught blue sharks (*Prionace glauca*) and mako sharks (*Isurus oxyrinchus*), lactate concentration was proposed as a useful predictor of mortality (Moyes *et al.*, 2006; Marshall *et al.*, 2012). Maximal plasma lactate concentrations recorded in the present study (<4 mmol/l) were much lower than those recorded for both surviving (5.80 ± 2.96 and 16.7 ± 12 mmol/l) and moribund (22.72 ± 4.07 and 34.3 ± 5 mmol/l) blue sharks and mako sharks, respectively (Moyes *et al.*, 2006; Marshall *et al.*, 2012). We recognize that lactate thresholds are species specific and linked to anaerobic capacity. Our results were more closely aligned with recent research on *H. portusjacksoni* and *M. antarcticus* that show no clear plasma lactate threshold leading to mortality in sharks (Frick *et al.*, 2010a).

#### Glucose

Changes in plasma glucose varied across the treatment groups. The fastest response was observed in the air-exposed group, and both the crowding and the extended trawl duration caused significant increase in glucose concentration. This change was not evident until 24 and 48 h after stress, respectively. Hyperglycaemia is recognized as a response to stress in elasmobranchs, with immediate increases in glucose attributed to mobilization of glycogen stores in the liver in reaction to the secretion of catecholamine, while delayed increases have been associated with the conversion of lactate to glucose as part of the anaerobic metabolism (Cliff and Thurman, 1984; Skomal and Mandelman, 2012). The sharp increase in glucose concentration observed in the air-exposure group may be attributed to the above phenomenon, because air exposure also induced a large change in lactate concentration. The delayed increase in plasma glucose in response to the 3 h trawl and crowd treatment is difficult to attribute to metabolized lactate, because these treatments induced relatively small changes in lactate concentration. Therefore, further investigation into the role of glucose production and removal as part of the physiological stress response is required in order to gain a better understanding of the consequences for stingaree homeostasis.

#### Urea

Urea concentrations continually decreased over the 48 h following trawling in response to air exposure, extended trawl duration and crowding. Decreased urea concentration can be indicative of osmotic imbalance or increases in urea permeability over the epithelial surface of the gills. This has previously been associated with the acute stress response in elasmobranchs and has been identified as an indicator of capture stress in spiny dogfish (*S. acanthias*; Mandelman and Skomal, 2009). Furthermore, decreases in urea concentrations can reflect an osmoregulatory response to a disturbed osmotic balance caused by a shift in fluid from extracellular to intracellular compartments, driven by the evident increase in intracellular lactate levels (Mandelman and Skomal, 2009). The continued decrease of urea concentration is indicative that urea may be a promising indicator of acute stress in stingarees; however, differences between treatment and control groups were only apparent 6 h after stress. This delayed reaction of urea concentration is further evidence for the importance of measuring blood metabolite concentrations for an extended time after stress, because samples taken directly after capture may underestimate the physiological impact of a capture event.

#### Potassium

Plasma potassium levels in response to crowding and air exposure increased throughout the blood-sampling period. The large variability between stingarees makes it difficult to draw meaningful biological conclusions from this response; however, hyperkalaemia has previously been associated with cellular disruption due to lactacidosis and is reported in other elasmobranchs [*S. acanthias* (Mandelman and Farrington, 2007), *H. portusjacksoni*, *M. antarcticus* (Frick *et al.*, 2010b) and *C. obscurus* (Cliff and Thurman, 1984)].

The maximal plasma potassium levels recorded in this study (air-exposure 48 h sample, 5.57 ± 0.52 mmol/l) were comparable to those recorded for *H. portusjacksoni* (5.69 ± 0.34 mmol/l) and *M. antarcticus* (5.73 ± 0.24 mmol/l) subjected to experimental trawls (Frick *et al.*, 2010b). Potassium has recently been found to serve as a potential indicator of mortality in long-line-caught sharks (Marshall *et al.*, 2012). The plasma potassium concentrations in the present study are much lower than the reported threshold for myocardial disruption of 7 mmol/l (Mandelman and Farrington, 2007), suggesting that potassium concentration did not reach levels likely to cause the death of stingarees. The reported threshold for myocardial disruption might not be applicable to all species, because *U. paucimaculatus* trawled for 30 min has shown maximal plasma potassium levels of 9.34 ± 0.32 mmol/l, without any links to mortality (Thomas and Chick, 2007).

#### Granulocyte-to-lymphocyte ratio

There was no significant difference in the G/L ratio between the control and the treatment groups; however, G/L ratio did increase over time. The largest G/L ratio increases were recorded in response to the 1 h trawl (649.77 ± 102.38%) and air-exposure treatments (266.76 ± 30.18%). Increases in the numbers of granulocytes (granulocytosis) in the blood can result from the trafficking of these cells into the peripheral blood to defend against foreign cells within the bloodstream (Dhabhar *et al.*, 1996). Decreases in the numbers of lymphocytes (lymphopenia) in the circulating blood results from their redistribution from the blood to the epidermis (Bonga, 1997). Granulocytosis and lymphophenia can indicate a change in immune strategy. This immunological response has previously been induced through long-line and gillnet capture of swellsharks (*C. laticeps*), and similar increases in G/L ratio were attributed to severe capture stress (Van Rijn and Reina, 2010). Granulocytosis is often associated with inflammation, which could be induced by physical damage during the experimental trawl or by the puncture wound caused by the blood sampling. We did not observe any differences in physical damage between treatments (M. Heard, personal observation) and it is, therefore, difficult to determine what effect this may have had.

### Air exposure

The results of this study suggest that compounded air exposure markedly increases the stress response of stingarees following trawl capture. While lactate levels changed significantly in all of the treatment groups, the magnitude of the change gives the best indication of the degree of stress that the animal has experienced (Elsasser *et al.*, 2000; Barton, 2002). Both initial and maximal observed lactate in the air-exposure group were more than twice as high as the respective lactate levels recorded in the 1 h trawl group and almost five times the respective lactate levels recorded for the control group. Increases in plasma lactate in response to aerial exposure have previously been reported in the little skate (*Leucoraja erinacea*), with increasing durations of air exposure causing larger changes in lactate concentrations (Cicia *et al.*, 2012). Additionally, increases in lactate levels in Atlantic sturgeon (*Acipenser oxyrinchus*) have previously been linked more closely to air exposure than to interaction with trawl gear (Beardsall *et al.*, 2013).

Air exposure led to an increase in glucose concentration and a decrease in urea concentration. Cicia *et al.* (2012) also reported hyperglycaemia in response to aerial exposure in *L. erinacea*, although the response was not consistent across seasons. Decreases in plasma urea concentrations have been previously linked to the stress response in spiny dogfish (*S. acanthius*; Mandelman and Farrington, 2007), with the magnitude of the change being similar to that observed in the present study. Additionally, increases in the G/L ratio (266.76 ± 30.18%) in response to air exposure, while variable, indicate potential changes in the immune strategy associated with the stress caused by a capture event. The magnitude of this change is comparable to those recently reported for stressed *C. laticeps* and is reflective of the severity of the stress induced in this study (Van Rijn and Reina, 2010).

The combination of high maximal lactate, increased glucose and decreased urea suggests that air exposure is the most physiologically detrimental factor of the capture process. These results support the findings of a recent study on *C. laticeps* that found air exposure to be the primary cause of physiological stress in a capture event (Van Rijn, 2009). Additionally, in several studies on marine teleosts [Pacific halibut (*Hippoglossus stenolepsis*; Davis and Schreck, 2005), lingcod (*Ophiodon elongates*; Milston *et al.*, 2006) and sablefish (*Anoplopoma fimbria*; Davis *et al.*, 2001)], it was also found that exposure to air evoked a significant stress response that was evident by significant elevations in plasma lactate.

### Trawl duration and crowding

Changing trawl duration and crowding induced changes across some of the parameters measured. While mortality was highest as a result of increased trawl time (37.5%), there were few differences in blood constituents between the 1 and the 3 h treatments, indicating that the increased trawl duration did not lead to an increase in the stress response. Our results corroborate recent field-based research, which recorded the immediate physiological response of stingarees to different trawl durations (15, 30 and 45 min) and found that while plasma lactate and glucose concentrations were significantly higher in the trawled stingarees in comparison to baseline concentrations, they were not significantly affected by increased trawl time (Thomas and Chick, 2007). Frick *et al.* (2010b) highlighted the difficulty in comparing results for trawl duration across studies due to the wide spectrum of methodological approaches and species studied. We used similar experimental protocols to those of Frick *et al.* (2010b), who found that there was no clear link between increased trawl duration for *M. antarcticus* and *H. portusjacksoni*. However, it is important to note that in a commercial trawl, increased trawl duration could lead to increased stress and mortality through the compounding of numerous factors, such as compression and hypoxia from increased catch size. For example, commercial trawls in the South Australian Prawn Trawl Fisheries commonly have combined catch rates (bycatch and target catch) of between 150 and 200 kg/h, which are likely to compound the negative effects of increased trawl time (Dixon *et al.*, 2005).

Effects of trawl net crowding, simulated by placing five stingarees in the cod-end at once, caused some additional stress response in stingarees in this study; however, the response was not of the same magnitude as that to air exposure. While these results are consistent with recent studies on other elasmobranch species, e.g. *H. portusjacksoni* and *M. antarcticus* (Frick *et al.*, 2010b) and *C. laticeps* (Van Rijn and Reina, 2010), we recognize that the conditions simulated in this study may not comparable to those experienced in a commercial trawl capture, where large quantities of catch, bycatch and abiotic material are netted (Dixon *et al.*, 2005). Mandelman and Farrington (2007) found that larger catches in Northwest-Atlantic demersal trawlers led to increased immediate and delayed morality in *S. acanthius.* Enever *et al.* (2009) also found a correlation between larger catch sizes and higher mortality rates for skates caught by demersal trawlers. Replication of the same level of crowding as that during normal fishing operations is logistically difficult in laboratory conditions, but the present study was nevertheless able to show that crowding increases the stress and physiological response of stingarees even though crowding was minimal. It is likely that crowding during commercial fishing operations magnifies the effects shown here.

### Conclusions

Our results indicate that stingarees are measurably affected by trawl capture and air exposure. This is evident from the elevations of physiological indicators of stress and post-capture mortality in this study, indicating that this species is not able to endure the severe stressors involved in trawl capture. We recognize that, with the small sample sizes in the present study, we were unable to differentiate which stressor caused the highest mortality; however, this is an important avenue for future research. Furthermore, mortality rates in the wild may be considerably higher where stingarees are subjected to more traumatic capture stressors and post-capture influences, such as temperature changes and predation. While the crowded treatment in the present study may have caused some form of additional density stress, it is unlikely to be comparable to the stress experienced by stingarees caught in commercial fishing operations.

We identified plasma lactate concentration as a useful indicator of stress for stingarees and found that peak levels were reached in the first 6 h following capture. In addition, plasma urea and glucose levels may be useful indicators of stress for stingarees, but levels did not change until 2 and 24 h post-capture, respectively. These factors support the findings of previous studies that repeated blood sampling is required to assess physiological stress in elasmobranchs (Cliff and Thurman, 1984; Frick *et al.*, 2009; Van Rijn and Reina, 2010). Characterization of the profiles of physiological indicators of stress will enable more accurate sampling of elasmobranchs in post-capture physiological studies by knowledge of the timing of peak responses in blood metabolites and ions. The physiological changes identified in this study reduce the need for repeated blood sampling and suggest that delayed sampling provides a more accurate assessment of secondary stressors in stingarees. We also found that leucocyte profiles can provide insight into the immunological response of stingarees to capture stress.

The large array of changes across all of the physiological stress indicators that were induced by an experimental trawl shows that air exposure is the most stressful element of a trawl capture event. The magnitude of the physiological changes caused by exposure to air in this study highlights the importance of returning stingarees to the water as quickly as possible after capture to increase their chance of survival. Management measures must take into account the potential for sublethal effects of capture, delayed reaction to stress and delayed mortality when assessing fishing bycatch mortality of stingarees. On-vessel measurements of bycatch mortality may underestimate the real effect of trawling operations if post-capture mortality is not considered.

## Funding

Funding was provided though a Flinders University Honours Scholarship to M.H. and Australian Research Council Linkage Grant LP110200572 to R.R.
